# Material Choice and Structure Design of Flexible Battery Electrode

**DOI:** 10.1002/advs.202204875

**Published:** 2022-11-20

**Authors:** Xiangling Xia, Jack Yang, Yang Liu, Jiujun Zhang, Jie Shang, Bin Liu, Sean Li, Wenxian Li

**Affiliations:** ^1^ School of Materials Science and Engineering Shanghai University Shanghai 200072 China; ^2^ Materials and Manufacturing Futures Institute School of Materials Science and Engineering The University of New South Wales Sydney NSW 2052 Australia; ^3^ College of Sciences Institute for Sustainable Energy Shanghai University Shanghai 200444 China; ^4^ Shaoxing Institute of Technology Shanghai University Shaoxing 312000 China; ^5^ School of Materials Science and Engineering Fuzhou University Fujian 350108 China; ^6^ Ningbo Institute of Materials Technology and Engineering Chinese Academy of Sciences Ningbo 315201 China

**Keywords:** deformable materials, deformable structure, energy storage, flexible electronics, wearable devices

## Abstract

With the development of flexible electronics, the demand for flexibility is gradually put forward for its energy supply device, i.e., battery, to fit complex curved surfaces with good fatigue resistance and safety. As an important component of flexible batteries, flexible electrodes play a key role in the energy density, power density, and mechanical flexibility of batteries. Their large‐scale commercial applications depend on the fulfillment of the commercial requirements and the fabrication methods of electrode materials. In this paper, the deformable electrode materials and structural design for flexible batteries are summarized, with the purpose of flexibility. The advantages and disadvantages of the application of various flexible materials (carbon nanotubes, graphene, MXene, carbon fiber/carbon fiber cloth, and conducting polymers) and flexible structures (buckling structure, helical structure, and kirigami structure) in flexible battery electrodes are discussed. In addition, the application scenarios of flexible batteries and the main challenges and future development of flexible electrode fabrication are also discussed, providing general guidance for the research of high‐performance flexible electrodes.

## Graphical abstract

Flexible electrodes play a key role in the energy density, power density, and mechanical flexibility of batteries. This review summarized the flexible battery electrodes from two key factors: flexible materials and flexible structures, aiming to achieve optimal flexibility from the functionalization of flexible materials and structural design.

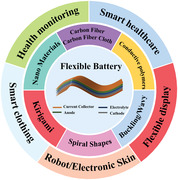

## 1 Introduction

Attributed to the development of the Internet of Things (IoT), flexible electronic devices have become critical components in flexible displays,^[^ ^1^ ^]^ robots,^[^ ^2^ ^]^ medical monitoring,^[^ ^3^ ^]^ and electronic skins^[^ ^4^ ^]^ in the most recent years. Independent power supply systems (batteries) are necessary for most commercial wearable devices (ear‐plugs, watches, and wristbands) to work. Series of batteries have been developed as self‐powered wearable devices, such as those that utilize wireless power supply,^[^ ^1^ ^]^ piezoelectricity,^[^ ^2^ ^]^ triboelectricity,^[^ ^3^ ^]^ and even biomass fuel cells.^[^ ^4^ ^]^ However, the output power from these energy sources is often lower than the normal operating power required for the key electronic components, such as Bluetooth^[^ ^3^, ^5^ ^]^ (>10 mW), Wi‐Fi^[^ ^6^ ^]^ (>20 mW), and some low‐power microcontroller unit (MCU)^[^ ^7^ ^]^ (>5 mW). Self‐powering can only serve as a secondary energy supply in certain equipment that requires the maintenance of stable operations for a long period. For example, a power shutdown of the equipment for monitoring the physiological signals from the human body may cause adverse consequences for diagnosing health conditions.^[^ ^8^ ^]^

Contributed to the good deformability and impact resistance, flexible batteries can be easily integrated into personal clothing, tents, packaging, and other objects as power supplies.^[^ ^21^ ^]^ Besides, the advantages of higher specific capacitance, excellent rate performance, flexibility, and high stability under mechanical deformation will be maintained while flexible batteries were adapted to their working conditions. Flexibility is not a strictly defined mechanical concept because all materials can be bended to some degree when their thicknesses are small enough. In this regard, the maximum bending amplitude (*f*) is often used to measure the flexibility of material without the occurrence of fracture and plastic deformation,^[^ ^22^ ^]^ which is defined as^[^ ^23^ ^]^

(1) $$f=\frac{2\sigma }{E\cdot d}$$

where *σ* is the yield strength of the material, *E* is the elastic modulus of the material, and *d* is the thickness of the material. Therefore, in addition to using low‐modulus materials to prepare flexible batteries, they generally occur as thin films with small thicknesses or wire structures with small radius, in order to maintain flexibility.

**Scheme 1:**
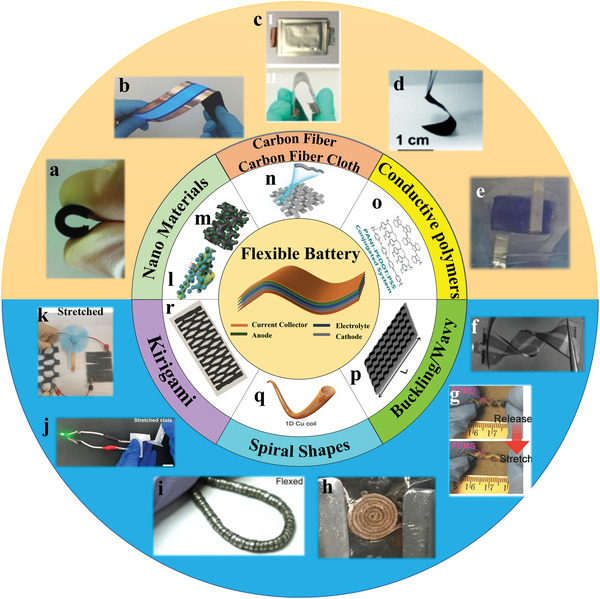
An overview of flexible electrodes based on flexible materials and flexible structures. Optional flexible materials include nanomaterials (carbon nanotubes [CNTs], graphene, MXene, etc.), carbon cloth, and conducting polymers. Optional flexible structures include buckling structures, spiral structures, and kirigami structures. The construction of the flexible battery and some flexible battery examples are also included. a) Reproduced with permission.^[^ ^9^ ^]^ Copyright 2012, Wiley‐VCH. b,n) Reproduced with permission.^[^ ^10^ ^]^ Copyright 2017, Wiley‐VCH. c) Reproduced with permission.^[^ ^11^ ^]^ Copyright 2016, Elsevier. d,l) Reproduced with permission.^[^ ^12^ ^]^ Copyright 2017, Royal Society of Chemistry. e,m) Reproduced with permission.^[^ ^13^ ^]^ Copyright 2017, Wiley‐VCH. f,p) Reproduced with permission.^[^ ^14^ ^]^ Copyright 2012, Wiley‐VCH. g) Reproduced with permission.^[^ ^15^ ^]^ Copyright 2017, Wiley‐VCH. h,q) Reproduced with permission.^[^ ^16^ ^]^ Copyright 2018, Elsevier. i) Reproduced with permission.^[^ ^17^ ^]^ Copyright 2017, American Academy for the Advancement of Science. j,r) Reproduced with permission.^[^ ^18^ ^]^ Copyright 2019, American Chemical Society. k) Reproduced with permission.^[^ ^19^ ^]^ Copyright 2020, American Chemical Society. o) Reproduced with permission.^[^ ^20^ ^]^ Copyright 2019, American Chemical Society.

Similar to conventional rigid batteries, flexible batteries consist of current collectors, cathode/anode electrodes, and electrolytes. Electrodes play a key role in the capacity, energy density and power density of batteries by supplying ions and electrons, and conducting electricity.^[^ ^24^ ^]^ The options of electrode materials and battery structures are crucial for high‐performance flexible batteries.^[^ ^25^ ^]^ An overview of flexible materials and flexible structures adopted for flexible electrodes was shown in **Scheme** **1**. Nanomaterials (carbon nanotubes [CNTs], graphene, MXene, etc.), carbon cloth (CC), and conducting polymers were the most common materials used as electrode materials for flexible batteries. Buckling, spiral, and kirigami structure were often used to construct flexible batteries.

However, the potential application scenarios of flexible batteries usually require thinness, strong deformability, and high deformation cycle stability, which brings new requirements for mechanical‐electrical properties, including battery capacity retention, voltage output stability, cyclic stability of structural and electrical properties in the case of mechanical deformation. Therefore, it is necessary to introduce new strategies to establish flexible electrodes, such as the preparation of flexible electrodes based on elastic/flexible materials and the design of flexible electrode structures. Recently, the research of flexible electrode materials and structures has been reviewed, from the types of electrode materials to the flexible battery devices. Li et al.^[^ ^26^ ^]^ reviewed paper‐based electrodes for flexible energy storage devices. Luo et al.^[^ ^27^ ^]^ discussed the future and challenges of flexible fiber batteries. Zhang et al.^[^ ^28^ ^]^ critically reviewed the latest developments in graphene‐based electrodes for flexible batteries. Fu et al.^[^ ^29^ ^]^ reviewed the application of carbon materials such as carbon nanotubes and carbon fibers (CFs) in thin film and fiber bendable flexible batteries. Qian et al.^[^ ^30^ ^]^ summarized the flexible structural design of lithium‐ion batteries (LIBs) for electronics and wearable devices. Xiang et al. explained the fundamental mechanisms underlying the structural design mechanism and intrinsically deformable materials as building blocks for flexible batteries including neutral plane design, serpentine patterning, and island architecture.^[^ ^31^ ^]^ Fu et al. discussed the requirements for constituent components, including the current collector, electrolyte, and separator, in flexible batteries including lithium‐ion batteries, non‐lithium‐ion batteries, and high‐energy metal batteries.^[^ ^29^ ^]^

We summarize recent review articles and experimental studies as shown in **Table** **1**. The fabrication of flexible electrodes is summarized into two strategies, i.e., depositing active materials on flexible substrates or converting rigid electrodes into flexible structures. From the perspective of material preparation, flexible batteries can be fabricated by preparing and synthesizing new flexible electrode materials (bottom‐up), i.e., depositing active materials on flexible substrates. From another perspective, the rigid material can obtain certain flexibility or stretchability through structure design, in order to achieve flexibility/elasticity in the final battery (top‐down). These two strategies are not completely independent. The strategy of developing new flexible/elastic electrode materials focuses on the intrinsic electrochemical properties of materials, and the improvement of the stretchable and bendable mechanical properties is relatively limited. The latter strategy is to introduce flexible structures into the electrode shape design, which can greatly improve the flexibility of the device. The common flexible structures contain fractals, paper cutting and textiles. There will be a loss of energy density for the strategy of flexible structure design due to the decline of space utilization and no improvement of the inherent electrochemical properties of materials. Therefore, to gain high‐performance flexible electrodes, the two strategies should be combined.

**Table 1:** Electrochemical performance and deformability of flexible battery electrodes

		Electrochemical performance		Deformability		
No.	Material/Structure	Specific capacity	Cycling performance	Maximum deformation	Deformation cycle	Ref.
1	CNT/Carbon nanofiber	370.8 mA h g^−1^ (at 200 mA g^−1^)	150.4 mA h g^−1^ (at 5 A g^−1^, 5000 cycles)			[32]
2	PDMS‐CNT	190 mA h g^−1^	160 mA h g^−1^ (at 15 cycles)	*r* ^a)^ = 4.5 mm		[9]
3	Fe_3_O_4_/CNT	758 mA h g^−1^ (at 100 mA g^−1^)	898 mA h g^−1^ (at 100 mA g^−1^, 100 cycles)	*r* ^a)^ ≈10 mm	200	[12]
4	Graphene‐modified PDMS sponge	126 mA h g^−1^ (at 50 mA g^−1^)	107.1 mA h g^−1^ (at 400 mA g^−1^, 300 cycles)	*ε* ^b)^ = 50%	100	[13]
5	Graphene‐based tin dioxide composite	870.8 mA h g^−1^ (at 100 mA g^−1^)	836 mA h g^−1^ (at 100 mA g^−1^, 100 cycles)			[33]
6	MXene‐bonded hard carbon	269.7 mA h g^−1^ (at 200 mA g^−1^)	272.3 mA h g^−1^ (at 200 mA g^−1^, 1500 cycles)	*r* ^a)^ ≈ 5 mm		[33]
7	Co_0.85_Se@Carbon mask fibers	1130 mA h g^−1^ (at 100 mA g^−1^)	841.33 mA h g^−1^ (at 100 mA g^−1^, 50 cycles)			[34]
8	Carbon‐cloth–carbon‐fiber	≈203 mA h g^−1^ (at 0.5 mA cm^−2^)	≈106.55 mA h g^−1^ (at 5 mA cm^−2^, 2400 cycles)	*r* ^a)^ ≈ 3 mm		[35]
9	Carbon cloth	121.6 mA h g^−1^ (at 0.125 mA cm^−2^)	69.5 mA h g^−1^ (at 1.0 mA cm^−2^, 200 cycles)	deg^c)^ = 90°	200	[11]
10	Ultrathin Co_3_O_4_/CC	542 mA h g^−1^ (at 2 mA cm^−2^)		*r* ^a)^ = 13 mm	300	[10]
11	PEDOT:PSS‐induced self‐doped PANI	238 mA h g^−1^ (at 200 mA g^−1^)	113 mA h g‐1 (at 10 A g^−1^, 1500 cycles)	deg^c)^ = 150°		[20]
12	Polypyrrole	1064 mA h g^−1^ (at 0.1C)	848 mA h g^−1^ (at 0.1C, 20 cycles)	deg^c)^ = 140°	10	[36]
13	Buckled			*ε* ^b)^ = 30%	10 000	[37]
14	Buckled/Arched	129 mA h g^−1^ (at 35 mA g^−1^)		*ε* ^b)^ = 400%	1000	[38]
15	Buckled/Wavy	3.1 mA h cm ^−2^ (at 0.2C)	≈2.48 mA h cm^−2^ (at 0.2C, 100 cycles)	*ε* ^b)^ = 120%	60 @50%*ε*	[15]
16	Spiral/Helical	1.2 mA h cm ^−1^ (at 0.5C)	1.128 mA h cm^−1^ (at 0.5C, 100 cycles)	*r* ^a)^ = 5 mm	17 000	[17]
17	Spiral/Helical	1 mA h cm^−2^ (at 1 mA cm^−2^)	176 cycles	*ε* ^b)^ = 60%	100	[16]
18	Kirigami: design 1	154.2 mA h g^−1^ (at 0.3C)	50 cycles	*ε* ^b)^ = 100%	500	[18]
	Kirigami: design 2	156 mA h g^−1^ (at 0.3C)	50 cycles	*ε* ^b)^ = 30%	500	

^a)^ Minimum bending radius

^b)^ Maximum tensile strain

^c)^ Maximum bending angle.

This review presents two strategies for fabricating flexible electrodes. The advantages and disadvantages of the flexible electrodes prepared in two strategies in terms of electrical properties (energy density, rate capability) and mechanical properties (failure under cyclic deformation conditions) are discussed and summarized respectively. We hope to provide instructive suggestions for the research and development of flexible electrodes through this review.

## 2 Deformable Materials for Electrode

The research of flexible electrode materials that can be applied to wearable devices focuses on stretchable materials with a bending radius of less than 30 mm and a tensile strain of more than 5% to fulfill the requirements of the PC‐2292 standard.^[^ ^39^ ^]^ Low resistance is also should be possessed for the electrode material to improve charge transfer efficiency.^[^ ^40^ ^]^ The competitive candidates that can meet both requirements include metal nanowires/carbon nanotubes, graphene, and carbon fiber (cloth). These materials can contribute to excellent bendability in the final products, even when the strain level is reaching to more than 50%.^[^ ^13^, ^41^ ^]^ As such, we categorize the following discussion based on different electrode materials.

## 3 Carbon Nanotube

As 1D nanomaterials, CNTs have unique mechanical and electrical properties due to the nanoscale sizes and quantum effects. For example, the tensile strength of carbon nanotubes is as high as 63 GPa,^[^ ^42^ ^]^ and metallic carbon nanotubes can theoretically withstand a current density of 4 × 10^9^ A cm^−2^, which is 1000 times greater than that of copper wire.^[^ ^43^ ^]^ Studies have shown that the greater the electrical conductivity of the electrode, the better the charge–discharge rate performance of the battery.^[^ ^24^, ^44^ ^]^ Attributed to their extremely low density, comparable electrical conductivity, and inherent flexibility, CNTs are considered as ideal materials to synthesize flexible electrodes, which combine high conductivity comparable to metals and flexibility comparable to rubbers.^[^ ^43^, ^45^ ^]^ There are many ways to synthesize CNTs, including chemical vapor deposition,^[^ ^46^ ^]^ hydrothermal,^[^ ^46^, ^47^ ^]^ electrospinning,^[^ ^48^ ^]^ and template methods.^[^ ^46^, ^49^ ^]^ However, pure carbon nanotubes often exhibit weak reversibility and low Coulombic efficiency.^[^ ^50^ ^]^ By introducing phosphorus,^[^ ^51^ ^]^ germanium,^[^ ^52^ ^]^ Fe_3_O_4_,^[^ ^53^ ^]^ and other activities into the carbon material, the active reaction sites can be increased to improve the electrode capacity or rate performance.^[^ ^9^, ^12^, ^54^ ^]^

**Figure 1:**
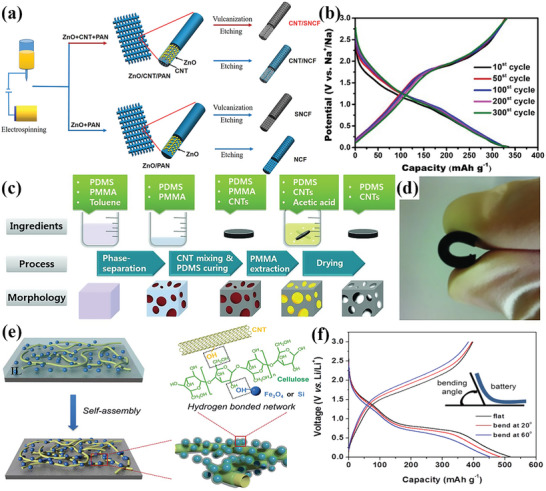
a) Schematic illustration for the synthesis of CNTs composite S/N co‐doped carbon nanofibers. b) Cyclic charge–discharge curves of CNT/SNCF electrodes at 0.2 A g^−1^. c) Process for preparing porous PDMS‐CNT nanocomposites. d) An example of a highly flexible PDMS‐CNT. e) Schematics illustrate the steps to fabricate the NP/CNT composites. f) Charge–discharge curves of electrodes at different bending angles (current density: 500 mA g^−1^). (a, b) Reproduced with permission.^[32]^ Copyright 2021, American Chemical Society. (c, d) Reproduced with permission.^[^ ^9^ ^]^ Copyright 2012, Wiley‐VCH. (e, f) Reproduced with permission.^[^ ^12^ ^]^ Copyright 2017, Royal Society of Chemistry.

S/N co‐doped carbon nanofibers/CNTs (CNT/SNCF) with a 3D network structure as electrode materials were synthesized by a electrospinning method by Chen et al.,^[^ ^32^ ^]^ as shown in **Figure** **1**a. The flexible electrode based on CNT/SNCF displayed good cycle (Figure 1b) and high discharge capacity (395.5 mA h g^−1^ at 0.1 A g^−1^). Porous polydimethylsiloxane (PDMS) material embedding with CNTs by removing polymethylmethacrylate (PMMA) from the PMMA‐PDMS and CNTs composite (Figure 1c) was applied as electrode material with highly flexibility (Figure 1d).^[^ ^9^ ^]^ The performance of the electrode benefits from the porous structure which contributed to a large specific surface area for contacting with the electrolyte, as well as a complex interconnecting structure as an efficient channel for ion diffusion.^[^ ^55^ ^]^ In another study, Fe_3_O_4_ nanoparticles were attached to the CNT surface through hydrogen bonding interactions between the cellulose neutralization and hydroxyl groups (—OH) on the surface of the active material to obtain high‐performance electrodes (Figure 1e).^[^ ^12^ ^]^ The Fe_3_O_4_/CNT flexible electrode showed good bendability, as shown in Figure 1f.

In addition to CNTs, metal nanomaterials (such as silver nanowires^[^ ^56^ ^]^ and copper nanowires^[^ ^57^ ^]^) are usually mixed with graphene, and CNTs, etc. as functional materials to improve electrical conductivity to prepare electrodes for flexible batteries. Metal oxide nanowires (such as SnO_2_, MnO_2_, and Co_3_O_4_) have high theoretical specific capacitances, but these materials are usually poorly conductive, and their volume expands and contracts significantly during charge and discharge. These materials need to be combined with other materials (usually carbon materials) when used.^[^ ^58^ ^]^

It should be emphasized that carbon nanotubes often play a role in enhancing both the electron transport and the surface area in traditional rigid battery electrodes,^[^ ^59^ ^]^ in which the intrinsic flexibility of carbon nanotubes is not critically needed. In recent years, the intrinsic flexibility of carbon nanotubes has been widely explored and applied in the field of flexible electronics such as flexible circuits and flexible batteries.^[^ ^60^ ^]^ In addition, carbon nanotube doping with active materials is another common approach to improve the performance of flexible electrodes.^[^ ^59^, ^61^ ^]^ However, there are a few remaining challenges for the usage of CNTs in flexible electrodes: 1) The overall performance of the CNTs is often unsatisfactory due to the contact resistance between particles, although individual CNT reveals good mechanical strength and electrical conductivity. The introduction of metal nanoparticles to form crosslinking effect is an approach to reduce the contact resistance, and the addition should be in the right amount to avoid material hardening which results in the loss of flexibility. 2) Electrodes composite of CNTs usually require binding aids or elastomer support to keep the CNTs network stable, which inevitably reduces the energy density of the battery. These problems can be further improved by optimizing the binder content or using conductive binders.^[^ ^62^ ^]^

## 4 Graphene and MXene

Graphene is a single layer of carbon atoms arranged in a two‐dimensional honeycomb lattice, which is an allotrope of graphite.^[^ ^63^ ^]^ Graphene is a high‐strength material with Young's modulus of 1 TPa and tensile strength of 130 GPa.^[^ ^64^ ^]^ Single‐layer graphene can be bent into a large angle while the resulting strain is almost negligible. The electrical conductivity of graphene is estimated to be as high as 1 × 10^5^ S cm^−1^.^[^ ^65^ ^]^ Graphene with a single layer will maintain excellent carrier mobility even in extreme deformation conditions.^[^ ^66^ ^]^ However, graphene materials, as a reinforcing material to increase Young's modulus and tensile strength of the composite material, are often mixed with other elastic materials as flexible electrodes. The addition of graphene will result in a certain loss of stretchability for the elastic material.^[^ ^67^ ^]^ Nonetheless, graphene is an ideal candidate for application in various flexible batteries, such as lithium‐sulfur batteries, metal‐air batteries, and metal‐ion batteries.^[^ ^28^, ^68^ ^]^

**Figure 2:**
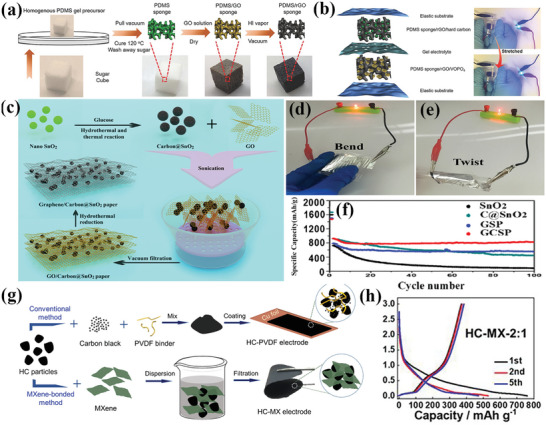
a) Schematic of conductive PDMS/rGO sponge preparation steps. b) Schematic diagram of the sodium‐ion full cell based on rGO sponge electrode (left) and photos of the working state of the cell before and after stretching (right). c) Schematic of the fabrication process of GCSP. Photograph of the normal operation of the flexible battery in the d) bent state and e) twist. f) Cyclic performances of SnO_2_, C@SnO_2_, GSP, and GCSP electrode (at 100 mA g^−1^). g) Schematic diagram of electrode preparation: (top) Conventional HC‐PVDF electrode. (bottom) HC‐MXene electrode. h) Charge/discharge performance of HC‐MX‐2:1 film at 30 mA g^−1^. (a, b) Reproduced with permission.^[^ ^13^ ^]^ Copyright 2017, Wiley‐VCH. c–f) Reproduced with permission.^[^ ^33^ ^]^ Copyright 2020, Elsevier. (g, h) Reproduced with permission.^[^ ^69^ ^]^ Copyright 2019, Wiley‐VCH.

The conductivity of the composite will be improved effectively by covering two‐dimensional layered graphene material on the surface of the elastic matrix. Graphene covered on porous PDMS was used as the flexible electrode by Li et al.^[^ ^13^ ^]^ as shown in **Figure** **2**a. The synthesized full cell consists of an elastic matrix encapsulation layer, a PDMS/rGO sponge electrode layer, and a gel electrolyte (Figure 2b). Benefiting from all the stretchable components, the flexible battery exhibited strain‐relaxation cycling stability and operation stably at a strain level of 50%. Thanks to the high electrochemical conductivity, stable porous structure, and strong mechanical deformability, the unique battery with the all‐stretchable component can be synthesized in quantity for the application of flexible/wearable/bendable devices power systems. Liu et al.^[^ ^33^ ^]^ studied how the incorporation of graphene incorporation affects the performance of the electrode, and they found that graphene contributes simultaneously both as a conductive material and a deformation‐inhibiting structure. The graphene/core–shell carbon@SnO_2_ paper (GCSP) was fabricated from carbon‐coated SnO_2_ (C@SnO_2_) dispersed into GO suspension, and the synthesis process was shown in Figure 2c. Benefiting from the stability of graphene‐coated SnO_2_ nanoparticles in large deformation and the inhibition of volume expansion of active material by graphene, the as‐prepared electrodes work stably in the bending state with high cycle stability, energy density, and high capacity retention of 836 mA h g^−1^ after 100 cycles with 0.04% capacity loss cycle^−1^ (Figure 2d–f).

Transition metal carbides and nitrides (MXenes) discovered in 2011 are another class of 2D inorganic compounds with high electrical conductivity, excellent ion mobility, good mechanical strength, and abundant surface functional groups.^[^ ^70^ ^]^ The applications of Mxenes in energy conversion and storage systems have been confirmed and reviewed in experimental and theoretical^[^ ^71^ ^]^ The application of MXene in flexible batteries also has been proved to be a promising direction.^[^ ^72^ ^]^ Numerous experimental and theoretical studies have shown that MXenes have exciting development potential in the fields of energy conversion and electrochemical storage. Here we look forward to the application of MXene in flexible battery electrodes.

Two types of hard carbon electrodes were prepared by a conventional MXene bonding method as shown in Figure 2g.^[^ ^69^ ^]^ The synthesis approach of the electrode is that the mixture of hard carbon (HC) and polyvinylidene fluoride (PVDF) binder is coated on the copper foil current collectors directly. In the MXene bonding process, HC particles are embedded into the 3D network of MXene sheet (HC‐MX) as the active material, eliminating the use of polymer binder and fluid collector. The electrode film based on HC‐MX with excellent flexibility and strength exhibits a specific capacity of 280.6 mA h g^−1^ and capacity retention of 100% after 300 charge–discharge cycles at a current of density 50 mA g^−1^. The results show the approval of the macroporous 3D structured MXene nanosheets applied in flexible batteries.

Although the applications of 2D materials (graphene, MXene) in flexible batteries have been confirmed, some barriers are still needed to overcome.^[^ ^28^, ^65^, ^72^, ^73^ ^]^ First, scaled‐up production of graphene or MXene with low cost and high quality is difficult.^[^ ^74^ ^]^ Second, the introduction of active materials will reduce the flexibility of graphene or MXene, thus it is necessary to optimize the mechanical and electrochemical properties to meet the demands of actual application. Finally, some problems still need to be solved, such as the poor tensile properties of graphene and MXene, and the degradation of electrochemical properties due to the easy oxidation of the MXene surface.^[^ ^28^, ^72^ ^]^

## 5 Carbon Fiber/Carbon Fiber Cloth

CFs composed of carbon atoms are a type of thread‐like material with diameters of 5–10 µm.^[^ ^75^ ^]^ The radius of carbon fiber is much larger than that of carbon nanotube, meaning reductions of the specific surface area as well as the bending ability for per unit mass CFs. Nevertheless, the production of carbon fiber with excellent bendability is simpler and easier compared with carbon nanotubes.^[^ ^76^ ^]^

**Figure 3:**
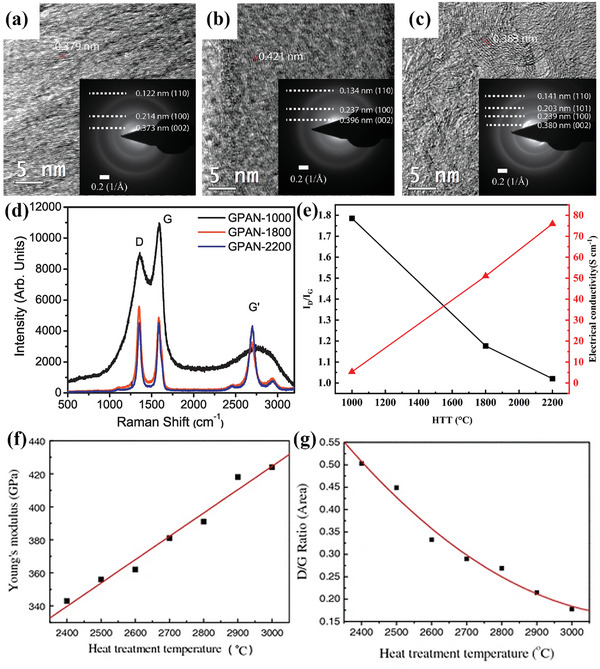
High‐resolution transmission electron microscope (HRTEM) images and selected area electron diffraction images (SAED, inset) of graphitized PAN fibers at a) 1000 °C, b) 1800 °C, and c) 2200 °C. d) Raman spectra of graphitized fibers at different temperatures. e) Raman peak intensity ratio (*I*_D_/*I*_G_) and electrical conductivity of carbon fibers at different heat treatment temperatures (data from ref. [77]). f) Effect of heat treatment temperature on Young's modulus of carbon fibers. g) Intensity ratio of D/G (area) as a function of heat treatment temperature. (a–d) Reproduced with permission.^[^ ^77^ ^]^ Copyright 2017, Elsevier. (f, g) Reproduced with permission.^[^ ^78^ ^]^ Copyright 2007, Springer.

The properties of carbon fibers vary greatly depending on the precursors and the heat treatment process, causing the different degrees of product graphitization in the carbonization process.^[^ ^79^ ^]^ As shown in **Figure** **3**a–e, the electrical properties of carbon fibers obtained by carbonization of PAN (GPAN) at different heat treatment temperatures (HTT) were investigated by Gupt et al.^[^ ^77^ ^]^ They find that the material resistivity tends to decrease with the increase of the graphitization of carbon fibers, and the resistivity decreases with an increasing degree of graphitization along the fiber axis due to the increase of the preferred orientation of the carbon layer.^[^ ^80^ ^]^ Li et al.^[^ ^78^ ^]^ studied the effect of heat treatment temperature on the modulus of PAN‐based carbon fibers, and found that the modulus of the fibers is proportional to the degree of graphitization of the carbon fibers, as shown in Figure 3f,g. The performances of PAN‐based carbon fibers and pitch‐based carbon fibers were discussed by Liu et al.^[^ ^81^ ^]^

**Figure 4:**
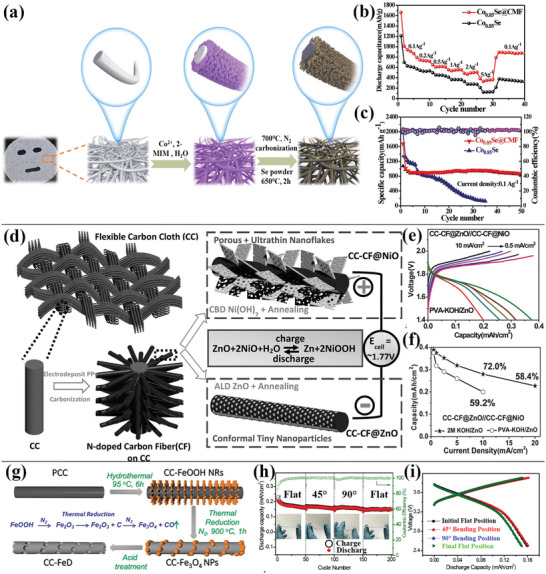
a) Process flow diagram for the preparation of Co_0.85_Se@CMF. b) Co_0.85_Se@CMF's charge and discharge curves at a current density of 0.1 A g^−1^. c) Rate performance of Co_0.85_Se@CMF and Co_0.85_Se at varied current densities. d) Schematic illustration of the preparation of N‐doped CFs on carbon cloth using metal oxides (NiO and ZnO) as electrodes for flexible Ni‐Zn batteries. e) Charge–discharge curves of flexible Ni‐Zn full batteries. f) Rate performance of the Ni‐Zn full cell. g) Fabrication process diagram of N‐doped porous CC‐FeD. h) Cycling stability of the samples in different mechanical states (current density is 1.0 mA cm^−2^). i) Charge–discharge curves of CC‐FeD//CC‐LCO flexible LIBs in different mechanical states (flat, 45°, and 90° bending). (a–c) Reproduced with permission.^[^ ^34^ ^]^ Copyright 2018, Elsevier. (d–f) Reproduced with permission.^[^ ^35^ ^]^ Copyright 2016, Wiley‐VCH. (g–i) Reproduced with permission.^[^ ^11^ ^]^ Copyright 2016, Elsevier.

Generally, the activity of as‐prepared carbon fiber is low, needing modifications to improve the activity as an electrode.^[^ ^11^, ^82^ ^]^ Besides, there is a class of materials called carbon fiber cloth (CFC) or CC obtained by the carbonization of textiles.^[^ ^83^ ^]^ The preparations of flexible electrodes from carbon fiber material and carbon cloth are similar, as shown in **Figure** **4**. It is a good choice for CF/CFCs were usually modified with active materials (metal oxides/hydroxides,^[^ ^84^ ^]^ metal sulfides,^[^ ^85^ ^]^ transition metal derivatives^[^ ^86^ ^]^) to improve their pseudocapacitive behavior, facilitating charging and discharging capabilities.^[^ ^82^, ^87^ ^]^ In addition, the large specific surface area of the CF/CFC composed of nanofibers^[^ ^84^, ^88^ ^]^ and nanosheets^[^ ^85c^ ^]^ will improve the electrical performance of flexible batteries.

N‐doped MOF‐derived composite Co_0.85_Se@Carbon Mask Fibers (Co_0.85_Se@CMF) on carbon substrate of compressed mask carbon fiber was applied as a flexible electrode by Yanan et al.,^[^ ^34^ ^]^ as shown in Figure 4a–c. The electrode can work normally under large‐angle bending, indicating that the carbon fiber has a good prospect to be used in the flexible electrodes. Importantly, there were no metal current collectors (such as Cu, Al film) in the flexible electrode based on Co_0.85_Se@CMF, maintaining a high energy density of 1130 mAh g^−1^. Liu et al.^[^ ^35^ ^]^ used another strategy to radially grow N‐doped CFs on each fiber of carbon cloth, and then deposited ZnO nanoparticles and NiO nanosheets on the CFs as anode and cathode, respectively (Figure 4d). The flexible full‐cell displayed a good rate capability of about 59.2% at 0.5 mA cm^−2^, as shown in Figure 4e,f. Carbon fibers could be further modified by introducing hollow structured in the material.^[^ ^11^, ^89^ ^]^ Porous surface nitrogen‐doped CC (CC‐FeD) was obtained by template method in the work of Balogund et al.,^[^ ^11^ ^]^ and the synthesis process was shown in Figure 4g. The introduction of micropores into CC possesses a 44‐fold increase of rate capacity compared with the pristine CC (PCC), and the as‐prepared fully flexible battery exhibits good cycling performance and similar discharge capacities of 0.159, 0.160, and 0.150 mA h cm^−2^ at flat, 45°, and 90° bending positions, respectively (Figure 4h,i).

**Figure 5:**
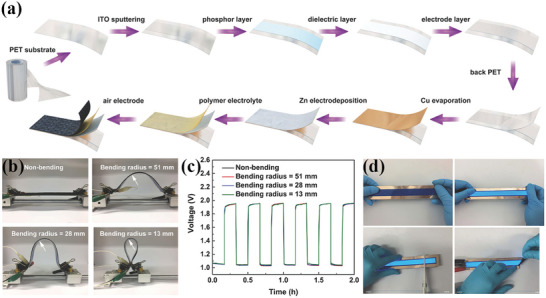
a) Schematic diagram of the fabrication process of a flexible display device integrated with an electroluminescent device and a flexible zinc‐air battery. b) Photographs of the flexible display device under various bending radius of the photo at different bending radius (flat, 51, 28, and 13 mm). c) Galvanostatic charge–discharge tests of flexible Zn‐air batteries at different bending radius (flat, 51, 28, and 13 mm). d)The flexible display device remained functional during the destruction process. Reproduced with permission.^[^ ^10^ ^]^ Copyright 2017, Wiley‐VCH.

The improvement of the safety performance and excellent flexibility of the batteries based on CC by the introduction of metal oxide nanoparticles has been demonstrated by Chen et al.^[^ ^10^ ^]^ Co_3_O_4_/CC air electrode was obtained from layered Co(OH)_2_ growing in situ on the surface of carbon fibers in carbon cloth (Co(OH)_2_/CC) by electrodeposition method with annealing treatment. **Figure** **5**a shows a flexible display unit integrated with a zinc‐air battery and a flexible display device, consisting of a zinc‐deposited copper film, a hydrogel electrolyte, and a Co_3_O_4_/CC air electrode. The final device shows a high degree of flexibility (minimum bending radius 13 mm, Figure 5b) and a negligible effect of bending on the battery performance (Figure 5c). The flexible battery can work normally with the imposition of shearing force (Figure 5d), indicating that the flexible battery has extremely high safety and stability and can be used in harsh environments or endure a certain degree of damage.

Carbon fiber and carbon cloth are very suitable substrates for flexible electrodes due to their good flexibility and self‐supporting, reducing the use of metal collectors and binders. The introduction of nanosized active materials into micrometer‐sized carbon fibers forms a multilevel microporous structure, which will increase the specific surface area of the material. Nanostructures can also shorten ion diffusion channels, accelerate the exchange of electrons and ions in electrolytes in electrode reactions, and greatly improve the energy density and rate capability of flexible batteries.^[^ ^90^ ^]^ Furthermore, it is difficult to control the stability of nanostructured carbon fibers due to the agglomeration caused by the larger surface energy of nanosized particles, which damages the long‐term stability of the materials and devices.

## 6 Conductive Polymers

Conductive polymers can also be used in flexible electronic devices.^[^ ^91^ ^]^ For example, heteroatom‐free polyacetylene (PAC),^[^ ^92^ ^]^ polyaniline (PANI),^[^ ^92^ ^]^ polyparaphenylene vinylene (PPV),^[^ ^93^ ^]^ N‐heteroatom‐containing polypyrrole (PPy),^[^ ^94^ ^]^ S‐heteroatom‐containing polythiophene (PT),^[^ ^95^ ^]^ poly(3,4‐ethylenedioxythiophene) (PEDOT),^[^ ^96^ ^]^ and poly(3,4‐ethylenedioxythiophene) polystyrene sulfonate (PEDOT:PSS)^[^ ^20^ ^]^ with a certain degree of conductivity and flexibility have been confirmed to be ideal materials for flexible electronic applications.^[^ ^97^ ^]^ However, there are some deficiencies of these polymer materials need to be solved.

**Figure 6:**
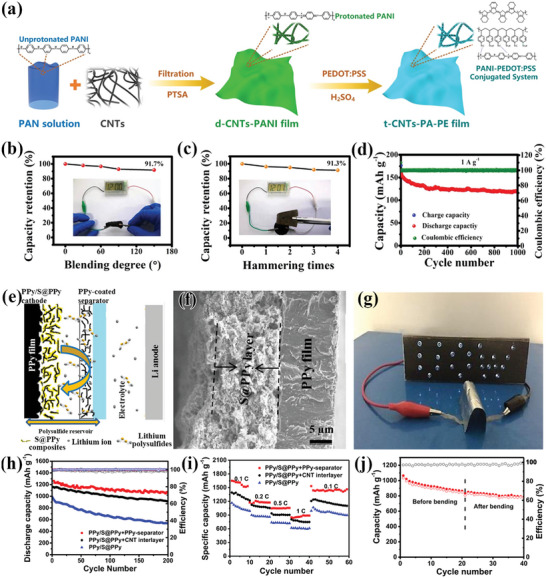
a) Schematic diagram of the fabrication of t‐CNTs‐PA‐PE. b) Capacity retention of ZIB at different bending angles. c) Capacity retention rate of ZIB under different hammering times. d) Cyclic performance of ZIB (at 1 A g^−1^). e) Schematic illustration of Li‐S battery fabricated with PPy/S@PPy electrode. f) Scanning electron microscope cross‐sectional images of PPy/S@PPy. g) Li‐S battery composed of PPy/S@PPy+PPy separator structure powers LEDs in the bent state. h) Cycling performance and i) rate performance of Li‐S batteries with different interlayer structures (PPy/S@PPy, PPy/S@PPy+PPy‐separator, and PPy/S@PPy+CNT interlayer) at 0.2 C. j) Cycling performance of the soft‐packaged battery at 0.1 C. (a–d) Reproduced with permission.^[^ ^20^ ^]^ Copyright 2019, American Chemical Society. (e–j) Reproduced with permission.^[^ ^36^ ^]^ Copyright 2018, Elsevier.

The spontaneous deactivation by deprotonation of PANI during charge and discharge would result in severe performance degradation.^[^ ^98^ ^]^ Construction of a *π*‐electron conjugated system between PANI and PEDOT: PSS on CNTs proposed to improve the electrochemical reaction stability of PANI‐based cathodes by Liu et al.,^[^ ^20^ ^]^ and the synthesis process was shown in **Figure** **6**a. To improve the electrical conductivity, the flexible film was treated with *p*‐toluene sulfonic acid (PTSA) solution in the fabrication and the final production (PEDOT:PSS coating on CNTs‐PANI film, CNTs‐PA‐PE) for zinc‐ion batteries (ZIBs) was obtained by soaking in H_2_SO_4_. The as‐prepared solid‐state ZIBs based on the CNTs‐PA‐PE showed good stability in bending and hammering tests with a capacity retention rate of 90%, and good cycling durability with capacity retention of 70% (118 mA h g^−1^) even after 1000 cycles at a current of 1 A g^−1^ (Figure 6b–d).

PPy synthesized by the oxidative chemical polymerization and electrodeposition method was used as electrodes and separators for flexible lithium‐sulfur batteries by Li et al.^[^ ^36^ ^]^ The cathode of the flexible batteries is prepared by applying a mixture of S@PPy, carbon black, and PVDF onto a PPy film (PPy/S@PPy), and the full cell is encapsulated by PPy/S@PPy, PPy separator, electrolyte, and lithium foil (PPy/S@PPy+PPy‐separator, Figure 6e,f). As shown in Figure 6g, the as‐fabricated cell demonstrates the ability to work under bending deformation. Contributed to the low charge transfer resistance of PPy separator, the as‐prepared full‐cell exhibited good flexibility, excellent capacity retention, and rate performance, as shown in Figure 6h,i. The working stability test of the battery under deformation conditions is shown in Figure 6j. After 20 cycles of discharge (0.1 C), the battery was bent ten times and then continued the test under the same conditions. Compared with the battery before bending, the battery almost maintained the same capacity, proving that the flexible Li‐S battery is of high stability.

Conducting polymers can be applied in a wide range of flexible electronics, including flexible wires, flexible supercapacitors, and fully flexible battery electrodes attributed to the electrical conductivity, mechanical flexibility, and optical properties.^[^ ^101^, ^102^, ^103^ ^]^ However, there are some drawbacks to the application of conductive polymer in flexible devices that need to be overcome. First, the conductivities of these polymers are low.^[^ ^99^ ^]^ Second, the diversion of polymer products caused by the polymerization mechanism and the difference of components due to the synthesize condition increases the difficulty to maintain the production uniformly.^[^ ^100^ ^]^

## 7 Challenges behind the Deformable Materials

**Figure 7:**
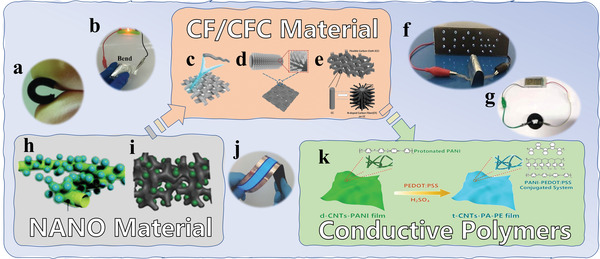
Flexible electrode materials and applications. a) Reproduced with permission.^[^ ^9^ ^]^ Copyright 2012, Wiley‐VCH. b) Reproduced with permission.^[^ ^33^ ^]^ Copyright 2020, Elsevier. c,j) Reproduced with permission.^[^ ^10^ ^]^ Copyright 2017, Wiley‐VCH. d) Reproduced with permission.^[^ ^82c^ ^]^ Copyright 2020, Wiley‐VCH. e) Reproduced with permission.^[^ ^35^ ^]^ Copyright 2016, Wiley‐VCH. f) Reproduced with permission.^[^ ^36^ ^]^ Copyright 2018 Elsevier. g,k) Reproduced with permission.^[^ ^20^ ^]^ Copyright 2019, American Chemical Society. h) Reproduced with permission.^[^ ^12^ ^]^ Copyright 2017, Royal Society of Chemistry. i) Reproduced with permission.^[^ ^13^ ^]^ Copyright 2017, Wiley‐VCH.

In the above discussions, the application of CNTs, graphene, MXene, CF/CFC, and conductive polymers in flexible electrodes was revealed. Although 1D nanomaterials based on carbon nanotubes have high flexibility and conductivity, curing agents are required in the synthesis process of flexible electrodes, which will damage the energy density of the flexible batteries. 2D nanomaterials (graphene and MXene) are promising candidates for flexible electrode materials. However, the high production costs and the difficult trade‐offs between mechanical and electrochemical properties limit the applications of 2D nanomaterials in flexible batteries. The use of the natural network structure of carbon fiber cloth can reduce or even avoid using binders and current collectors. The main issue of CFC in the prepared flexible electrode is to improve electrical conductivity. The development of nanomaterials for flexible electrodes is shown in **Figure** **7**. Mixing conductive nanomaterials (such as carbon nanotubes and graphene) with elastic materials is a simple method, and flexible electrode materials with both flexibility and conductivity can be obtained by adjusting the content of conductive materials. The elastic material only plays the role of a deformable matrix and a binder reducing the mass‐specific capacitance of the overall electrode material. Therefore, the binder‐free flexible electrode based on CC appears more attractive except for the stretchability. The synthesis of flexible electrodes based on self‐supporting structured carbon cloth maintains the sufficient specific surface area and modification ability, abandoning collectors and binders. Conductive polymers may be good candidates for both stretchability and conductivity, avoiding binders and being stretchable.

In conclusion, carbon cloth materials are the preferred materials for current flexible electrodes, while conductive elastic polymers have natural stretchability superior to carbon cloth materials and seem to be good candidates for future flexible electrodes. Though the defect of low conductivity, conductive polymers are considered as the major development direction of future flexible electronic devices in some works.^[^ ^20^, ^101^ ^]^

## 8 Deformable Structure for Electrode

Structural design and engineering provide a critical strategy to fabricate flexible electrodes with rigid materials.^[^ ^102^ ^]^ The early approaches to flexible structures were obtained by simply thinning rigid materials, with the purpose of releasing the internal stress generated by the electrodes during deformation.^[^ ^103^ ^]^ Subsequently, novel strategies of structural designs, such as buckling structure,^[^ ^14^, ^104^ ^]^ island‐bridge structure,^[^ ^105^ ^]^ paper‐cutting (kirigami) structure,^[^ ^19^, ^106^ ^]^ and spirals structure^[^ ^107^ ^]^ have been adopted to gain large deformation in the structure. The application of various structural designs in flexible energy storage devices and flexible electronic products has been confirmed to be a good option.^[^ ^108^ ^]^

## 9 Buckling Structure

Buckling structure, known as wavy structure, usually refers to a curved surface structure like a wave obtained by pre‐straining. The physical strain of the buckling structure is accommodated through the modulation of amplitude and wavelength.^[^ ^109^ ^]^ The typical prestretching method is that: first, conductive materials/electrode materials were attached to a prestretched flexible substrate (e.g., PDMS,^[^ ^110^ ^]^ polyurethane [PU],^[^ ^111^ ^]^ and silicone elastomer prepolymer [Ecoflex]^[^ ^112^ ^]^), then releasing the strained substrate to achieve the stretchable product.^[^ ^113^ ^]^ However, this manufacturing method naturally limits the working range of the device, that is, the maximum strain amount cannot exceed the prestrain value.^[^ ^14^, ^104^, ^114^ ^]^

**Figure 8:**
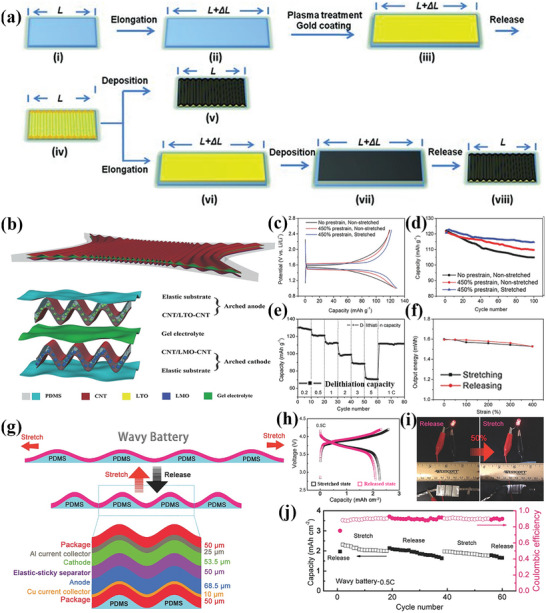
a) Schematic illustration of the fabrication of buckling Au or PPy‐pTS thin films on SIBS substrates. b) Schematic illustration of the superelastic battery fabricated from the arched electrode structure. c) Output voltage distribution and d) cycle life of CNT/LTO‐CNT composites are affected by prestretching and stretching treatments. e) Rate performance of CNT/LTO‐CNT composites after stretching cycles (400% stretching, 500 cycles). f) The effect of the areal mass density of LTO on the electrochemical performance of the CNT/LTO‐CNT composite. g) Schematic diagram of the composition structure and stretching–release of the wavy battery. h) Discharge/charge voltage curves of the wavy battery in stretched and released states. i) The wavy battery powers the LED in different states (release and 50% stretch). j) Cycle performance of wavy battery in different states (release and 50% stretch). (a) Reproduced with permission.^[^ ^37^ ^]^ Copyright 2011, Wiley‐VCH. (b–f) Reproduced with permission.^[^ ^38^ ^]^ Copyright 2015, Wiley‐VCH. (g–j) Reproduced with permission.^[^ ^15^ ^]^ Copyright 2017, Wiley‐VCH.

The synthesis process of a typical flexible electrode with a buckling structure is shown in **Figure** **8**a, and the applications of the flexible electrode in magnesium batteries demonstrate excellent mechanical stability in maintaining the electrochemical performance after 2000 stretching cycles at 30% strain.^[^ ^37^ ^]^ A unique arch‐structured stretchable LIB composed of CNTs and lithium manganese oxide (LMO) nanoparticle cathode, gel electrolyte, CNTs, and lithium titanium oxide (LTO) nanoparticle anode was designed by Weng et al.^[^ ^38^ ^]^ as shown in Figure 8b. The sandwich structured battery encapsulated with elastic polymer PDMS can operate at up to 450% strain state due to the prestrain of 450%. The as‐prepared exhibited an almost identical electrochemical performance with a capacity of 129 mA h g^−1^ (Figure 8c,d). The rate capability test (Figure 8e) after 500 stretching cycles and the output energy comparison of the battery under 400% strain (Figure 8f) further demonstrate good electrochemical performance and long‐life performance. An all‐wave‐shaped stretchable battery was designed and showed a nearly constant surface area capacity of about 2.2 mA h cm^−2^ in the released and stretched states by Liu et al.^[^ ^15^ ^]^

In general, two shortcomings limit the application of buckling structures. First, some tearing in the pre‐attached film may be generated because the active material expands perpendicular to the stretching axis during the strain release while the compressive deformation occurs in the direction perpendicular to the axis.^[^ ^104b^ ^]^ Second, the as‐prepared materials can maintain highly stable performance when working under the upper limit of pre‐strain, but irreversible performance loss may occur once the prestrain limit is exceeded.^[^ ^14^ ^]^

## 10 Spiral/Helical Shapes

**Figure 9:**
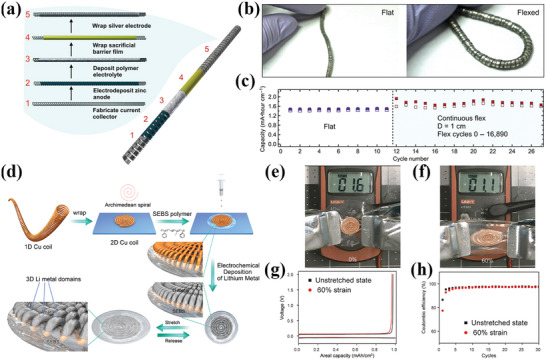
a) Schematic diagram of the structure of the helical ribbon spring‐shaped flexible battery. b) Photographs of the flexible wire battery in the flat and flexed state. c) Cycling performance of wire battery in flat and 1 cm bending diameter states. d) Schematic showing the fabrication process of the stretchable electrode. Photographs showing the stretchability of the stretchable electrode. Output voltage of the helical stretchable battery e) flat and f) 60% stretched. g) Voltage curves and h) cycling performance of helical stretchable lithium metal anodes in unstretched and 60% stretched states. (a–c) Reproduced with permission.^[^ ^17^ ^]^ Copyright 2017, American Academy for the Advancement of Science. (d–h) Reproduced with permission.^[^ ^16^ ^]^ Copyright 2018, Elsevier.

Spiral or helical shapes are generally made of conductive wire/tape wrapped around a forming model that can potentially act like a spring. Spiral shapes possess large stretchability attributed to the presence of multiple helical coils along the axial direction, which can distribute large deformation into small parts.^[^ ^115^ ^]^ This structure can be well applied to materials with poor stretchability (such as metal electrodes) to achieve good bending properties and stretchability.^[^ ^116^ ^]^ Zamarayeva et al.^[^ ^17^ ^]^ designed a flexible spiral structured Ag‐Zn batteries composed of an electro‐galvanizing anode, dip‐coating polyvinyl alcohol (PVA) electrolyte, wrapping cellophane on prefabricated tin‐coated copper spiral ribbon current collectors and silver cathode as shown in **Figure** **9**a. The helical silver‐zinc battery with a minimum bending diameter of 10 mm exhibits an initial specific discharge capacity of 1.33 mA h cm^−1^, and slight capacity fluctuations in the continuous working state of bending deformation (Figure 9b,c). It is believed that the increase in active surface area of the silver electrode caused by the bending deformation enhances the capacity of the flexible batteries. A flexible lithium metal anode based on a helical copper which can be stretched in a 2D plane was designed by Liu et al.^[^ ^16^ ^]^ (Figure 9d). The capacitance of the stretchable Li metal anode is about 1 mA h cm^−2^, and the voltage curves (Figure 9e–g) and cycling performance (Figure 9h) in the unstretched state and the 60% stretched state are not significantly different, confirming the good tensile stability of the electrode. In general, helical electrodes have spring‐like properties of large stretching or bending, which is a good choice for increasing the flexibility of rigid electrode materials.

## 11 Kirigami Structure

Applying the Kirigami structures is another strategy to achieve flexible electrodes with intrinsically inflexible materials. Through cutting the material into a specifically designed pattern, the Kirigami structure can effectively release the internal stress of the material through out‐of‐plane deformation. The device can maintain stable electrochemical performance when large deformation occurs.^[^ ^117^ ^]^ Some applications of Kirigami structures in flexible energy storage devices have been carried out, such as stretchable zinc‐air batteries^[^ ^19^ ^]^ and stretchable micro‐supercapacitor arrays.^[^ ^106a^ ^]^ It shows that the rational design of the Kirigami structure can be applied to flexible electrodes and even flexible electronic devices.^[^ ^117^ ^]^

**Figure 10:**
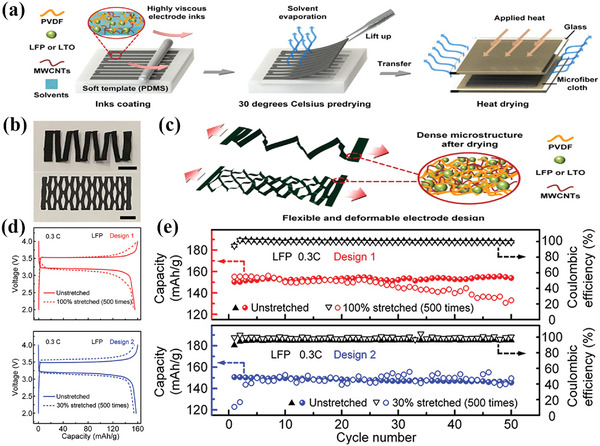
a) Schematic diagram of the fabrication process of the kirigami‐shaped electrode. b) Electrode photographs of two different kirigami structures. c) Schematic diagram of the deformation of electrodes with different kirigami structures under tension. The inset shows the electrode microstructure. d) Charge–discharge curves and e) cycle performance of flexible batteries fabricated with two kirigami structures. Reproduced with permission.^[^ ^18^ ^]^ Copyright 2019, American Chemical Society.

Taking the work of Bao et al. as an example, the synthesis process of the Kirigami electrode by using PDMS as soft templates is shown in **Figure** **10**a.^[^ ^18^ ^]^ In this work, two types of Kirigami structured electrodes were designed. The deformation of the two Kirigami electrodes under tension was tested, and the result indicated a good connection of the electrode in an out‐of‐plane deformation, maintaining the stability of the electrode (Figure 10b,c). The two as‐prepared Kirigami electrodes exhibited a constant unchanged discharge capacity after 500 times of 100% stretch–release cycles (Figure 10d). The cyclic charge–discharge performance tests of two kirigami designs unstretched and after 500 stretch–release cycles were shown in Figure 10e. Both electrodes display a stable discharge capacity and high Coulombic efficiency (close to 100%) for over 50 charge–discharge cycles in the unstretched states. The electrochemical performance of the two electrodes after 500 stretch–release cycles maintains high discharge capacities and coulombic efficiencies but little inferiority to the unstretched electrode.

## 12 Challenge of Deformable Structure

**Figure 11:**
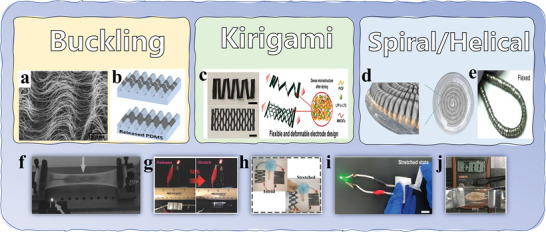
Development of flexible structures (top) and application examples (down). a) Reproduced with permission.^[^ ^14^ ^]^ Copyright 2012, Wiley‐VCH. b) Reproduced with permission.^[^ ^104b^ ^]^ Copyright 2013, IOP Publishing Ltd. c,i) Reproduced with permission.^[^ ^18^ ^]^ Copyright 2019, American Chemical Society. d,j) Reproduced with permission.^[^ ^16^ ^]^ Copyright 2018, Elsevier. e) Reproduced with permission.^[^ ^17^ ^]^ Copyright 2017, American Academy for the Advancement of Science. f) Reproduced with permission.^[^ ^38^ ^]^ Copyright 2015, Wiley‐VCH. g) Reproduced with permission.^[^ ^15^ ^]^ Copyright 2017, Wiley‐VCH. h) Reproduced with permission.^[^ ^19^ ^]^ Copyright 2020, American Chemical Society.

The fabrication processes of flexible electrodes through structural engineering (e. g. buckling, Kirigami, and spiral/helical) were reviewed in this section. More than 100% tensile can be obtained for poor tensile/bendable materials through these strategies, expanding the application potential of flexible structure design.^[^ ^38^ ^]^ A summary of several flexible electrode structures and their exemplary applications in batteries is shown in **Figure** **11**. Buckling structures are commonly used for thin‐film electrodes on elastic substrates; Kirigami structures can be used for most thin‐film/paper electrodes; helical structures make metal electrodes flexible and stretchable. In addition, there are many flexible structures, including island bridge structures (stretchable batteries composed of rigid battery “islands” and curved conductive “bridges”),^[^ ^105^ ^]^ fractal structures (looks like some kind of kirigami structure, but the pattern obeys fractal geometry, such as the Hilbert curve),^[^ ^118^ ^]^ and so on. The design of novel flexible structures has broad development space with the development of fine additive manufacturing technologies, such as 3D printing.

## 13 Application of Flexible Battery

**Figure 12:**
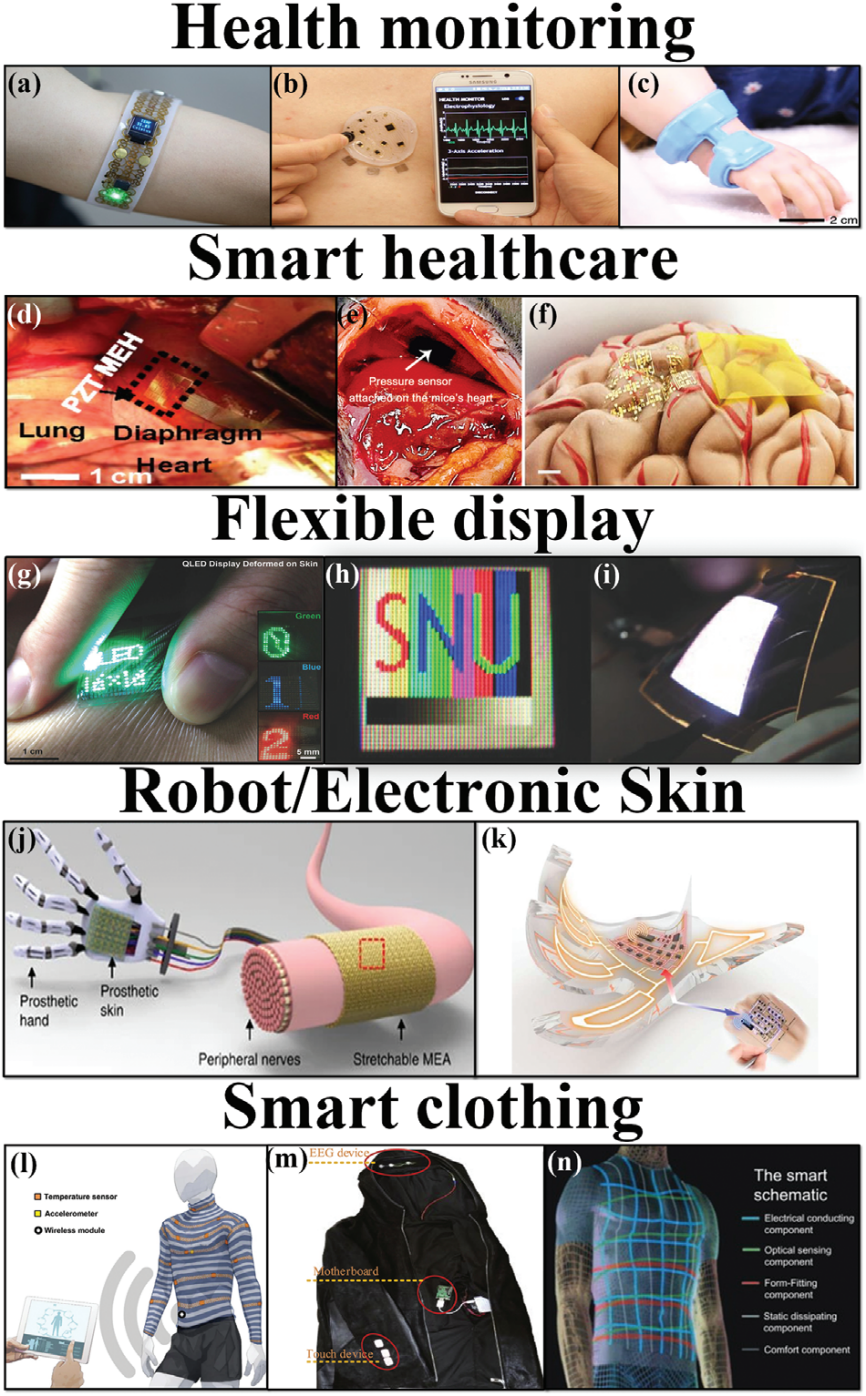
Potential applications of flexible batteries, including a–c) health monitoring, d–f) smart health care, g–i) flexible displays, j,k) robot/electronic skin, and l–n) smart clothing. (a) Reproduced with permission.^[125]^ Copyright 2019, American Association for the Advancement of Science. (b) Reproduced with permission.^[126]^ Copyright 2017, Springer Nature. (c) Reproduced with permission.^[127]^ Copyright 2020, Springer Nature. (d) Reproduced with permission.^[^ ^128^ ^]^ Copyright 2015, The Authors, published by National Academy of Sciences. (e) Reproduced with permission.^[129]^ Copyright 2021, Elsevier. (f) Reproduced with permission.^[130]^ Copyright 2017, The Authors, published by National Academy of Sciences. (g) Reproduced with permission.^[131]^ Copyright 2017, Wiley‐VCH. (h,i) Reproduced with permission.^[132]^ Copyright 2014, Wiley‐VCH. (j) Reproduced with permission.^[133]^ Copyright 2014, Springer Nature. (k) Reproduced with permission.^[134]^ Copyright 2018, American Academy for the Advancement of Science. (l) Reproduced with permission.^[135]^ Copyright 2020, Springer Nature. (m) Reproduced with permission.^[136]^ Copyright 2020, Elsevier. (n) Reproduced with permission.^[137]^ Copyright 2014, Elsevier.

Potential application scenarios of flexible batteries are highlighted in this section, including health monitoring, smart medical care, flexible display, robotics, electronic skin, and smart clothing (**Figure** **12**). The healthcare industry is a very promising target market. Flexible medical electronics can be applied in wearable devices, implantable medical devices, and interventional medical devices, as shown in Figure 12a–f. Wearable electronic devices can be used for daily health monitoring of the human body (heart rate and body temperature), and can also be used for medical monitoring (blood oxygen, blood flow).

The physiological state of the body (intracranial pressure, electrocardiogram, electrocorticography) can be accurately monitored by implantable/interventional flexible electronic devices, providing real‐time detection for treatment.^[^ ^119^ ^]^ Of course, it also can be directly used in medical treatment, such as cardiac pacemakers, controlled drug release, etc. Flexible batteries can be used as an energy‐supplying system for these devices requiring a long‐term stable operation to maintain real‐time monitoring of user/patient physiological information.

Integration with flexible display devices (Figure 12g–i) will be a broad market for flexible batteries. As a window for information transmission, the display device plays a vital role in the connection between software and hardware (such as rollable mobile phones and wearable smart bands/bracelets).^[^ ^120^ ^]^ In 2018, Rouyu Technology released the world's first fully flexible screen foldable smartphone and then set off a wave of foldable phones. Moreover, a report from International Data Corporation (IDC) predicts that the global production value of wearable devices will reach $106.35 billion by 2025. (Source: IDC Worldwide Quarterly Wearable Device Tracker, September 2021.) As a power supply device for such flexible display devices, flexible batteries have a huge market demand.

Another application area of flexible batteries is to power flexible electronics(robots and electronic skins) as shown in Figure 12j,k. Acquisition of surpassing human perception capabilities for robots is the goal for robotics and electronic skin.^[^ ^121^ ^]^ Moreover, flexible electronic skins and prostheses have been supplied for disabilities for a better quality of life.^[^ ^122^ ^]^ Flexible electronics can revolutionize robotics and prosthetics and bring about the next major revolution in the electronics industry. In robotics and related applications, flexible electronics are expected to revolutionize the way machines interact with humans, real‐world objects, and the environment.^[^ ^123^ ^]^ For example, an electronic skin on a robot's body would allow safe robotic interaction during physical contact with various objects.

Smart clothing (electronic textiles) is also a potential application of a class of flexible batteries, as shown in Figure 12l–n. Traditional textiles are only used as covering materials, while smart clothing can receive and respond to environmental stimuli. Smart clothing plays a key role in various technologies (communication, information, health‐care monitoring, military, sensors, magnetic shielding, etc.).^[^ ^124^ ^]^

In summary, flexible batteries as energy storage devices have broad application prospects by providing a stable and reliable power supply for flexible displays, flexible sensors, and other components. Different performance requirements are put forward for flexible batteries in different application scenarios.

## 14 Conclusion and Future Prospects

In this work, we reviewed two strategies to fabricate flexible battery electrodes, i.e., flexible materials‐based electrodes and flexible structures made of rigid materials. The main conclusions include:

(i) The flexible electrode is an essential part of flexible batteries, and their roles contain transporting electrons, providing electrode reaction interfaces, supporting battery structures, and realizing flexible properties. The flexible electrode material has a decisive influence on the battery's energy density, rate performance, and flexibility. The flexible structure design plays an important role in improving the mechanical properties of flexible batteries and broadening the application range. The research on flexible battery electrodes will promote the innovation of flexible electronics, and broaden the practical application of flexible electronics.
(ii) Carbon‐based elements or compounds are the most popular materials used as flexible electrodes. Carbon nanotubes, graphene, and MXene have superior flexibility, good electron conductivity, and mechanical strength as electrodes because of their unique microstructures and the C‐C bonding. The huge specific surface areas benefit to anchor the active materials to remedy the low energy density. However, the high cost of fabrication is a challenge for industrial‐scale applications. Carbon fiber and carbon fiber cloth are the perfect alternatives to the expensive carbon nanotube, graphene, and MXene to fabricate large‐scale flexible electrodes. Carbon fiber and carbon cloth are also self‐supporting flexible electrodes to improve power density. Quality control is one of the most challenging for carbon fiber and carbon cloth applications as flexible electrodes. Conductive polymers are potential candidates for flexible electrodes for electron conductivity and flexibility as well as possible optical transparency.
(iii) Combining flexible materials with flexible structures is an efficient strategy to improve the stretchability of batteries. Flexible electrodes can be made from rigid materials via structure design to absorb the energy of macromechanical deformation. Thin film, buckling, spirals, island‐bridge structure, and paper‐cutting (Kirigami) are all possible structures for flexible electrodes. The strategies of flexible structures and flexible materials are not entirely independent. However, this combined strategy also has a trade‐off between mechanical properties and energy density. Structures such as Kirigami and helical structures can achieve large apparent elongation rates but are accompanied by a rapid decrease in energy density per unit volume/area. Therefore, it is necessary to develop new deformable structures or design suitable structures according to practical applications to trade off mechanical and electrochemical properties.

Many flexible electrodes have been reported based on the materials and structure design, substantial room exists for the flexible electrode design with excellent cyclic stability, good mechanical performance, and high power and energy densities.

(i) Carbon‐based materials will still play a vital role in the development of flexible electrodes. Chemical stability, electrical conductivity, mechanical strength, and flexibility are the most competitive factors for carbon‐based materials to be employed as electrodes. Carbon‐based materials can be designed in different styles, from nanometers and micrometers to macroflexible materials. The growth or deposition of active material on carbon materials to improve their electrochemical properties is one of the challenges for carbon‐based materials.
(ii) Flexible polymer‐based electrodes are the other good choice for developing flexible batteries because of their light weight and possible transparency natures. Low electrical conductivity, low‐rate performance, low active material loading, and stability in electrochemical environments are the challenges for the application of conductive polymer in flexible batteries.
(iii) Most active materials used in flexible materials are inorganic and naturally rigid, which cannot be loaded or grown on carbon‐based and polymer‐based materials efficiently. The power density and energy density of flexible batteries are limited by the low content of active materials. Structures involving more active materials should be designed in the future to make the flexible devices work for a longer time. Furthermore, the growth or loading techniques also influence the stability of the flexible batteries.
(iv) Portable devices require thin and light electrodes as well as excellent mechanical properties, and active materials with high energy density are needed to develop. The mechanical performance depends on the connection of the active materials with the flexible substrates. Both active materials and flexible substrates suffer from the requirement of optical transparency for some devices.
(v) The fabrication of nanomaterials and nanostructures is always suffering from the energy‐consuming process of waste chemical disposal. New techniques should be developed to produce large‐scale and low‐cost flexible electrodes (carbon‐based and polymer‐based electrodes) in a simple and energy‐saving process. It is necessary to develop multifunctional electrodes to reduce the independent components in flexible batteries and improve the mechanical stability of flexible batteries.
(vi) Criterions for flexible battery electrodes should be constructed as soon as possible in order to provide a reference for researchers to evaluate the performance level of their prepared electrodes. The criteria need to be suitable for the mechanical and electrochemical properties of flexible electrodes to balance these two incompatible factors.

## Conflict of Interest

The authors declare no conflict of interest.

## Author Contributions

X.X.: conceptualization, investigation, writing – original draft; J.Y.: conceptualization, investigation, writing – original draft; Y.L: writing – original draft; J.Z.: conceptualization, writing – review & editing; J.S.: writing – review & editing, supervision; B.L.: funding acquisition, writing – review & editing; S.L.: conceptualization, writing – review & editing; W.L.: conceptualization, funding acquisition, supervision, writing – review & editing.
